# Environmental Surveillance for Polioviruses in Haïti (2017–2019): The Dynamic Process for the Establishment and Monitoring of Sampling Sites

**DOI:** 10.3390/v13030505

**Published:** 2021-03-18

**Authors:** Mary M. Alleman, Angela D. Coulliette-Salmond, Pierre Wilnique, Hanen Belgasmi-Wright, Leanna Sayyad, Kimberly Wong, Edmund Gue, Robert Barrais, Gloria Rey-Benito, Cara C. Burns, Everardo Vega

**Affiliations:** 1 Polio Eradication Branch, Centers for Disease Control and Prevention, Global Immunization Division, Atlanta, GA 30329, USA; 2 Polio and Picornavirus Laboratory Branch, Centers for Disease Control and Prevention, Division of Viral Diseases, Atlanta, GA 30329, USA; jrq0@cdc.gov (A.D.C.-S.); zqd1@cdc.gov (C.C.B.); ftt6@cdc.gov (E.V.); 3 United States Public Health Service, Rockville, MD 20852, USA; 4 Laboratory and Research, Division of Epidemiology, Ministère de la Santé Publique et de la Population (Ministry of Public Health and Population (MSPP)), Port au Prince HT6110, Haiti; wilniquep@yahoo.com (P.W.); mcrbarrais@yahoo.fr (R.B.); 5 IHRC, Inc., Atlanta, GA 30346, USA; ywp8@cdc.gov (H.B.-W.); nvj5@cdc.gov (K.W.); 6 Cherokee Nation Assurance, Catoosa, OK 74015, USA; pqd0@cdc.gov; 7 Pan American Health Organization, World Health Organization, Region of the Americas, Port au Prince HT6110, Haiti; edmondgue@paho.org; 8 Pan American Health Organization, World Health Organization, Washington, DC 20037, USA; reyglori@paho.org

**Keywords:** polio eradication, environmental surveillance, vaccine-derived polioviruses, wild poliovirus, enterovirus

## Abstract

Haïti is at risk for wild poliovirus (WPV) importation and circulation, as well as vaccine-derived poliovirus (VDPV) emergence. Environmental surveillance (ES) for polioviruses was established in Port au Prince and Gonaïves in 2016. During 2017–2019, initial ES sites were re-evaluated, and ES was expanded into Cap Haïtien and Saint Marc. Wastewater samples and data on weather, hour of collection, and sample temperature and pH were collected every 4 weeks during March 2017–December 2019 (272 sampling events) from 21 sites in Cap Haïtien, Gonaïves, Port au Prince, and Saint Marc. Samples were processed for the detection of polio and non-polio enteroviruses using the two-phase and “Concentration and Filter Elution” methodologies. Polioviruses were serotyped and underwent intra-typic characterization. No WPV or VDPVs were isolated. Sabin-like polioviruses (oral vaccine strain) of serotypes 1 and 3 were sporadically detected. Five of six (83%), one of six (17%), five of six (83%), and two of three (67%) sites evaluated in Cap Haïtien, Gonaïves, Port au Prince, and Saint Marc, respectively, had enterovirus isolation from >50% of sampling events; these results and considerations, such as watershed population size and overlap, influence of sea water, and excessive particulates in samples, were factors in site retention or termination. The evaluation of 21 ES sampling sites in four Haïtian cities led to the termination of 11 sites. Every-four-weekly sampling continues at the remaining 10 sites across the four cities as a core Global Polio Eradication Initiative activity.

## 1. Introduction

In 1988, the World Health Assembly passed a resolution to eradicate poliomyelitis (polio, caused by wild poliovirus (WPV) infection), a viral disease with an oral–fecal route of transmission [1,2,3,4]. Surveillance for cases of acute flaccid paralysis (AFP) in children and virologic testing is one of the four strategic pillars for eradication used by the Global Polio Eradication Initiative (GPEI); however, surveillance for AFP has low sensitivity for poliovirus infection, as paralysis occurs in <1% of poliovirus infections, which may limit the detection of poliovirus circulation in areas of low transmission [1,2,3]. Sensitivity of AFP surveillance may be diminished further if poorly implemented (i.e., the non-polio AFP rate is below the expected baseline of one per 100,000 population in people less than 15 years of age) [2,5]. Environmental surveillance (ES) for polioviruses, including the collection and analyses of wastewater/sewage for polioviruses, is increasingly being used to complement AFP surveillance [2,3,6,7,8,9,10,11,12,13,14,15,16,17]. The expansion of ES is a principal activity proposed in the GPEI’s Polio Endgame Strategy 2019–2023 [14]. ES has detected WPV and vaccine-derived poliovirus (VDPV) transmission in both the presence and absence of virus detection through AFP surveillance [2,6,7,8,9,10,11,12,13,18].

The ongoing efforts to characterize ES sampling sites, to optimize sensitivity for detecting poliovirus transmission, are critical, especially in the late stages of eradication [7,8,15,17]. In non-WPV-endemic countries, the consistent isolation of enteroviruses (e.g., Sabin-like (SL) vaccine strain polioviruses and non-polio enteroviruses (NPEVs)) in wastewater samples has been used as a working criterion that an ES system for polioviruses is sufficiently sensitive to detect WPVs and VDPVs [2,7,17].

In 2014, the Pan American Health Organization (PAHO) began to consider implementing ES in selected settings in the Americas to rapidly detect WPV circulation in the event of the importation and the emergence of VDPVs [17]. Haïti was considered a priority country for the establishment of ES, due to its (a) long history of sub-optimal polio vaccination coverage (estimated national coverage of the third dose of oral polio vaccine (OPV; Pol3) never exceeded 70% from 1980–2013), (b) poor hygienic and sanitary conditions, (c) high levels of population movement and high numbers of international visitors, and (d) history of a circulating VDPV type 1 (cVDPV1) outbreak in 2000–2001 [3,17,18,19,20,21]. In 2016, ES for polioviruses was established in two densely populated Haïtian cities: Port au Prince and Gonaïves [17,22,23]. The results of the first year of sampling (March 2016–February 2017) have been reported [17]. After that feasibility assessment, certain initial ES sites were re-evaluated, and ES was expanded into Cap Haïtien and Saint Marc, two additional densely populated cities [22,23]. Here we report the processes and methodologies used for the evaluation, expansion, and optimization of ES sampling sites during March 2017–December 2019.

## 2. Materials and Methods

### 2.1. Review of Estimated Annual National Pol3 Coverage

A review of Haïti’s estimated annual national-level Pol3 coverage was conducted with data available from 1980–2019 [20]. With the exception of 2019, when coverage was estimated at 74%, coverage for all years was ≤67%.

### 2.2. Environmental Surveillance Site Selection 

**Figure 1:**
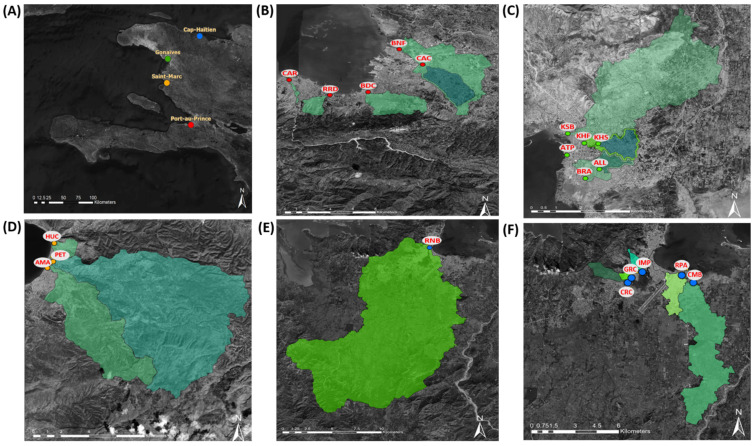
Haïti poliovirus environmental surveillance sites during March 2017–December 2019, site locations. (**A**) Location of the cities of Cap Haïtien, Gonaïves, Port au Prince, and Saint Marc. Poliovirus environmental surveillance sampling sites in (**B**) Port au Prince (BDC = Bois de Chêne, BNF = Bois de Neuf, CAC = Cite au Cayes, CAR = Carrefour, and RRD = Route Rails Diquini; the MAC site is not shown [17]); (**C**) Gonaïves: (ALL = Avenue Leon Legros, ATP = Autorité Portuaire, BRA = Boulevard de l’Avenir, KHF = Key Soleil Health Facility, KHS = Key Soleil School, and KSB = Key Soleil Bridge); (**D**) Saint Marc (AMA = Avenue Maurepas, HUC = Impasse Hucar, and PET = Rue Petion); (**E**) Cap Haïtien (RNB = Route National Bridge); and (**F**) Cap Haïtien (CRC = Ruelle Caporis, GRC = Grand Rue Champin, IMP = Impasse Petion, CMB = Rivière Commerce Bridge, and RPA = Ruelle Patience). Watershed boundaries for each sampling site are indicated in green shading, except for ATP, where the boundaries could not be determined [17,24].

A total of 21 sampling sites in four coastal cities—Cap Haïtien, Gonaïves, Port au Prince, and Saint Marc (Table 1, Figure 1A–F)—were selected and evaluated, considering GPEI environmental surveillance guidelines [2]. These four cities were chosen because they are among the most populated in the country with road accessibility throughout the rainy and dry seasons, are in regions involved in the 2000–2001 cVDPV1 outbreak, and have open canals that make sample collection feasible [17,18,19,22,23]. Maps for each city, where the catchment area and hydrology dynamics were derived from a 30m terrain model (digital elevation model), were used to localize the open canals and assist in the site selection process [24]. Geographic coordinates for each site were measured by a handheld GPS device (Montana 600; Garmin International, Olathe, KS, United States), and watershed populations were estimated from a WorldPop spatial demographic dataset (Department of Geography and Environment, University of Southampton, Southampton, United Kingdom) [24]. The watershed populations, corresponding to certain sampling sites, were smaller than recommended in the GPEI guidelines (>100,000 persons); however, these sites were evaluated for feasibility, due to limited options for safely accessible collection sites in the chosen cities [2].

### 2.3. Sample Collection and Frequency 

The Haïtian Ministère de la Santé Publique et de la Population (Ministry of Public Health and Population (MSPP)) project coordinator and sample collectors were trained on sample collection and personal protection procedures through classroom and field training [17]. Staff from the Centers for Disease Control and Prevention in Atlanta (CDC-Atlanta, GA, USA) and PAHO conducted twice-yearly field supervision visits during the surveillance period.

Two wastewater samples (1 L each) were collected from the 21 different sampling sites during 272 sampling events in Cap Haïtien (six sites, 60 sampling events), Gonaïves (six sites, 61 sampling events), Port au Prince (six sites, 110 sampling events), and Saint Marc (three sites, 41 sampling events), approximately every 4 weeks during March 2017–December 2019, except for September and October 2019, when sampling was not possible in Gonaïves and Saint Marc due to civil insecurity (Table 1, Figure 1A–F). Collection was conducted using the grab method, with a swing sampler (NASCO, Fort Atkinson, WI, USA) using 1 L Nalgene® bottles. Time and date of collection, sample temperature, and weather conditions on collection day and the previous day were recorded.

All samples were maintained in a 2–8 °C cold chain from collection until arrival at the Laboratoire National de Santé Publique (LNSP) in Port au Prince, where they were stored at −20 °C until shipment on dry ice to CDC-Atlanta. Upon arrival, samples were maintained at −20 °C until processing for virologic analyses.

### 2.4. Environmental Sample Processing

All samples were processed by the Polio and Picornavirus Laboratory Branch at CDC-Atlanta. Before processing, both 1 L environmental water samples from each sampling site were thawed at room temperature for 24 h, combined into a sterile glass beaker with a stir bar to mix for 15 min, and split into duplicate aliquots when parallel virus concentration methods were used. Samples collected during March 2017–November 2017 and July–August 2019 were processed using only the two-phase separation method, and those from September 2019–December 2019 were processed using only the “concentration and filter elution” (CaFÉ) methodology (described below) [2,17]. Samples collected during December 2017–June 2019 and samples collected in August 2019 at the Route National Bridge (RNB), Impasse Petion (IMP), and Rivière Commerce Bridge (CMB) sites in Cap Haïtien were processed in parallel with the two-phase separation method (500 mL) and the CaFÉ (500 mL) method. The remaining volume from each sampling site was refrozen at −20 °C in case re-testing was necessary. Enterovirus isolation results from the two methods are combined in the Results section; however, results from each method are provided separately in Supplementary Figure S1.

### 2.5. Two-Phase Separation Method

A volume of 500 mL was concentrated using the two-phase separation method, as described previously [17]. Antibiotics were added to the concentrate at 100 IU/mL penicillin, 100 µg/mL streptomycin, and 50 µg/mL gentamycin. The resulting concentrates were inoculated into cell culture for enterovirus isolation on the same day, as described below.

### 2.6. Concentration and Filter Elution Filtration Method

A volume of 500 mL was processed using the CaFÉ procedure, which utilizes a 1 L, stainless steel coffee press (VonShef, London, UK) using an 8 µm filter (85 mm, grade 1, Cytiva Life Sciences, Hillerød, Iceland). Briefly, wastewater was added to the carafe and then pressed to separate the sediment from liquid. Virus was extracted from the sediment by adding beef extract (3% *w*/*v*; Criterion, Hardy Diagnostics, Santa Maria, CA, USA) and chloroform–dithizone (0.001% *w*/*v*; Sigma-Aldrich, St. Louis, MO, USA) to a 50 mL conical tube containing the sediment and the filter. Samples were agitated for 5 min using a Heidolph (Schwabach, Germany) shaker and subsequently centrifuged at 1500× *g* for 10 min. The supernatant was transferred to the liquid from the original sample. Magnesium chloride hexahydrate (2.5% *w*/*v*; EMD Millipore Corp, Burlington, MA, USA) was added to the sample, and the pH was adjusted to 3.5.

The pressed filtrate was passed through a series of two additional filters. The first stage used a 5 µm filter with a diameter of 47 mm, made of mixed cellulose ester, and the second stage used a 0.45 µm filter with a diameter of 47 mm made of mixed cellulose ester (both from Advantec, Toyo Roshi Kaisha, Ltd., Uchisaiwaicho, Chiyoda City, Japan). Both filters were subsequently cut into four pieces and placed in a 50 mL conical tube containing beef extract (3% *w*/*v*) and agitated for 20 min.

The resulting concentrate was treated with chloroform (20% *v*/*v*), agitated for 20 min, and the phases were separated by centrifugation (1500× *g*, 20 min). Antibiotics (100 IU/mL penicillin, 100 µg/mL streptomycin, and 50 µg/mL gentamycin) were added to the supernatant. The resulting concentrates were inoculated into cells for enterovirus isolation on the same day, as described below.

### 2.7. Virus Isolation

Polioviruses, including SL, and NPEVs were isolated according to the recommended World Health Organization poliovirus isolation protocol, using cell cultures of L20B cells (recombinant murine cells that express human poliovirus receptor) and human rhabdomyosarcoma (RD) cells, followed by detection and intratypic characterization of polioviruses by real-time (RT)-PCR. [2,25].

## 3. Results

### 3.1. Sample Collection and Water Quality Analyses

For all sites in Cap Haïtien and Saint Marc; the Bois de Neuf (BNF), Bois de Chêne (BDC), and Route Rails Diquini (RRD) sites in Port au Prince; and the Boulevard de l’Avenir (BRA) and Key Soleil Health Facility (KHF) sites in Gonaïves, the median collection hour was before 10:00 a.m. (Table S1). For the remaining sites in Gonaïves and Port au Prince, the median collection hour was between approximately 10:00 a.m.–2:00 p.m. By site, the percentage of sample collection events with rain on the prior day and on the day of collection ranged from 0–50% and 0–8%, respectively. By site, median sample temperatures ranged between approximately 26–33 °C, and median pH values ranged between 6.6–7.4.

### 3.2. Enterovirus Detection

**Figure 2:**
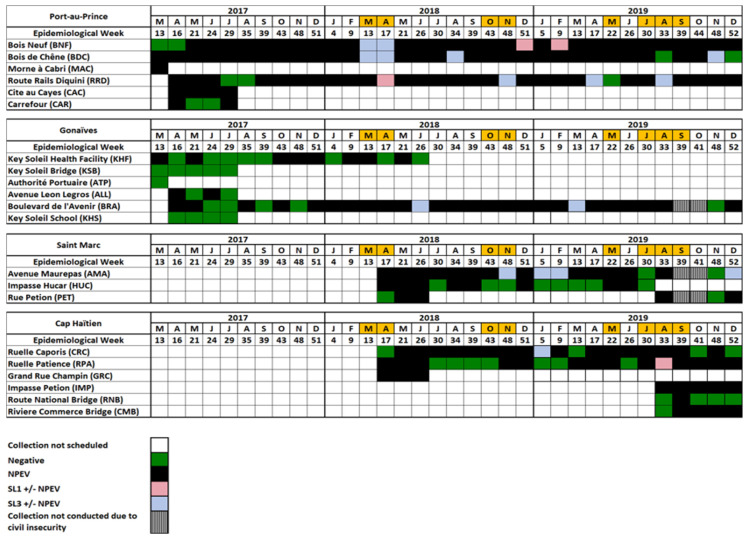
Enteroviruses isolated through Haïti poliovirus environmental surveillance during March 2017–December 2019 by year, epidemiological week, and sampling site. Orange shading for months in 2018 and May 2019 indicates periods of tetanus–diphtheria outbreak response campaigns in certain regions, which provided an opportunity for intensified all-antigen vaccination, and for July–September 2019 indicates the approximate time period of a nationwide supplementary immunization activity targeting children 2–59 months of age with bivalent oral polio vaccine (bOPV, containing Sabin strain serotypes 1 and 3). NPEV = non-polio enterovirus; SL1 = Sabin-like poliovirus of serotype 1; SL3 = Sabin-like poliovirus of serotype 3.

No WPVs or VDPVs were isolated from any sample (Figure 2 and Figure S1).

#### 3.2.1. Port Au Prince

No SL polioviruses were isolated from any sample collected during March 2017–February 2018 (Figure 2 and Figure S1). SL polioviruses of serotypes 1 and 3 were isolated from samples collected at the BNF, BDC, and RRD sites in various months between March 2018–November 2019; each site had four instances of SL poliovirus detection, with serotype 3 being the most frequently isolated (9 of 12 collections). NPEV and/or SL polioviruses were isolated from collections during 91–100% of sampling events at the BNF (32/34), BDC (32/34), Morne à Cabri (MAC) (1/1), RRD (30/33), and Cite au Cayes (CAC) (4/4) sites, and during 50% of sampling events at the Carrefour (CAR) site (2/4) (Figure 2, Table 2, and Figure S1).

#### 3.2.2. Gonaïves

No SL polioviruses were isolated from any sample collected between March 2017–May 2018 (Figure 2 and Figure S1). SL poliovirus of serotype 3 was isolated from samples collected from the BRA site in June 2018 and March 2019. NPEV and SL polioviruses were isolated from collections during 50–84% of sampling events at the KHF (8/16), Avenue Leon Legros (ALL) (2/4), and BRA (26/31) sites and during 0% of sampling events at the Key Soleil Bridge (KSB) (0/5), Autorité Portuaire (ATP) (0/1), and Key Soleil School (KHS) (0/4) sites (Figure 2, Table 2, and Figure S1).

#### 3.2.3. Saint Marc

SL poliovirus of serotype 3 was isolated from four samples collected from the Avenue Maurepas (AMA) site between November 2018–December 2019 (Figure 2 and Figure S1). NPEV and SL polioviruses were isolated from collections during 17/19 (90%), 8/16 (50%), and 4/6 (67%) sampling events at the AMA, Impasse Hucar (HUC), and Rue Petion (PET) sites, respectively (Figure 2, Table 2, and Figure S1).

#### 3.2.4. Cap Haïtien

SL poliovirus of serotype 3 was isolated from a sample collected from the CRC site in January 2019, and an SL poliovirus of serotype 1 was isolated from a sample collected from the Ruelle Patience (RPA) site in August 2019 (Figure 2 and Figure S1). NPEV and SL polioviruses were isolated from collections during 67–100% of sampling events at the Ruelle Caporis (CRC) (17/21), RPA (14/21), Grand Rue Champin (GRC) (3/3), IMP (5/5), and CMB (4/5) sites, and during 20% (1/5) of sampling events at the RNB site (Figure 2, Table 2, and Figure S1).

## 4. Discussion

ES for polioviruses was established in Haïti for the timely detection of WPV circulation after importation and the emergence of VDPVs [17]. During the surveillance period reported here (March 2017–December 2019), no WPVs or VDPVs were detected in any environmental sample collected in four of the country’s most populated cities (Cap Haïtien, Gonaïves, Port au Prince, and Saint Marc) [22,23]. Moreover, neither had been detected in Port au Prince or Gonaïves during the previous surveillance period, March 2016–February 2017 [17].

Because OPV replicates in the intestines of recipients and is excreted in feces, SL polioviruses can be detected through ES [2,7,15,16,17,18]. Haïti’s routine immunization schedule for polio provides bivalent OPV (bOPV1,3, serotype 1- and 3-containing) at birth, 10 and 14 weeks, and nine months of age [26]. Between May 2016–February 2018, no SL polioviruses were isolated from any ES site [17]. Subsequently, between March 2018 and December 2019, there was sporadic isolation of SL polioviruses of serotypes 1 and 3 in each of the four cities, with the isolation of serotype 3 being more common; of the 20 total occurrences of SL poliovirus isolation, 16 (80%) were serotype 3 and 12 (60%) were from sites in Port au Prince. The more frequent isolation of SL polioviruses in Port au Prince over time, compared to the other cities, could be due to the greater estimated watershed populations associated with the ES sites, or to greater access to vaccination services, as it is the capital city. A temporal pattern of SL poliovirus isolation was not obvious; however, isolations could be related to instances of intensified vaccination activities, when large numbers of primary vaccinees and their close contacts are shedding SL polioviruses simultaneously. During the surveillance period, there was a nationwide bOPV1,3 supplemental immunization activity from 15 July–5 September 2019, targeting children 2–59 months of age [27]. Moreover, from March–April and October–November 2018 and May 2019, there were tetanus–diphtheria outbreak response campaigns in certain Haïtian regions that provided opportunities for all-antigen vaccination of under-vaccinated and un-vaccinated children [27]. During 2017–2019, there were 31 AFP cases notified in Haïti; only one case, in 2019, had SL poliovirus (of serotype 3) isolated from stool. Infrequent isolation of SL polioviruses from ES sites, with consistent NPEV isolation, and from AFP cases was also noted in Haïti during March 2016–February 2017 [17]. Similar infrequent SL isolations through ES have been observed in other OPV-using countries; the reasons are not well-characterized [6,28,29]. Haïti’s national-level Pol3 coverage delivered through routine services was estimated at 64-74% annually during 2016–2019 [20]; actual coverage among the ES site watershed populations might be significantly lower than these estimates. Alternatively, the fecal matter of children in the target age for routine immunization services may not be well-represented in the open canals from which samples are collected.

SL poliovirus of serotype 2 was last isolated in Haïti through ES in Port au Prince in March 2016 [17]; none was isolated from any ES sample during the current surveillance period. In April 2016, Haïti participated in the global switch, during which the use of trivalent OPV (tOPV, serotype 1-, 2-, and 3-containing) ceased and was replaced with bOPV1,3 [12,16]. The absence of SL poliovirus of serotype 2 in Haïti’s ES samples collected during this surveillance period provides support for a successful switch and an absence of the post-switch, type 2 circulating VDPV outbreaks that have been observed in other countries [11,12,13,16].

Most ES sample collection during the surveillance period was conducted before 10:00 a.m.; exceptions were collection events at certain sites in Port au Prince and Gonaïves where the median collection times were much later than the typical peak time frame for the flushing of human sewage [2,3]. Later-than-recommended collection times might have been irrelevant for the MAC site, a Port au Prince wastewater treatment facility, but could have negatively impacted the presence of enteroviruses at the other sites. Field-measured sample temperatures and laboratory-measured sample pH did not suggest conditions that would adversely affect enterovirus survival. Median laboratory-measured pH values for samples from all Haïti’s ES sites have been in the neutral range over time [17]. An attempt was made to measure physico-chemical properties (i.e., pH, total dissolved solids, conductivity, and salinity) of samples at the sampling sites using a hand-held device; however, values were erratic, making interpretation difficult (data not shown). Other investigators have succeeded with such field-measured parameters and found that pH ≥8.5 and concentrations of total dissolved solids ≥1.77 mg/L were independently associated with higher rates of enterovirus isolation [7].

The consistent isolation of enteroviruses over time, in conjunction with other considerations and estimated watershed populations, was used to determine the retention or termination of the 21 ES sites assessed in Haïti during March 2016–December 2019 (Table 2) [17]. The primary reason for terminating collection at eight sites in the four cities (CAR in Port au Prince; KHF, KSB, ATP, ALL, and KHS in Gonaïves; HUC in Saint Marc; and RNB in Cap Haïtien) was an enterovirus isolation rate of ≤54% during the combined periods assessed, between March 2016–December 2019 (Table 2) [17]. Sampling from the MAC site in Port au Prince was discontinued after March 2017 due to the difficulty of processing the samples because of their high levels of particulate matter and to inability to identify the geographic origins of the populations contributing to the waste [17]. The CAC site in Port au Prince, upstream from BNF, provided promising results; however, BNF is preferred, due to its downstream location capturing a greater watershed and its performance over time [17]. After three months of collection with satisfactory enterovirus isolation at GRC, it was nonetheless terminated due to a very low estimated watershed population size (5445 persons).

A total of ten sites in the four cities were retained (Table 2). The major population areas of Port au Prince are covered by ongoing collection at three sites—BNF, BDC, and RRD—that span the coast of the city from east to west, respectively (Figure 1B). The four sites retained in Cap Haïtien represent watersheds from populations in the western (CRC and IMP) and eastern (CMB and RPA) areas of the city (Figure 1F). The PET and AMA sites in Saint Marc span the coast from north to south (Figure 1D).

## 5. Conclusions

This report describes the dynamic process of establishing and monitoring ES sites for polioviruses. The analyses of data from many months of sample collection are often necessary to make evidence-based judgements of site performance and to inform decisions for terminating or retaining sites and exploring new ones [7,17]. The experience with ES in Haïti continues to inform the GPEI as it expands ES implementation [14]. Because of longstanding suboptimal poliovirus vaccination in Haïti, the country remains at high risk for the circulation of any imported WPV and the emergence and circulation of VDPVs [20]. Attention is needed towards strengthening the performance of Haïti’s AFP surveillance system; non-polio AFP rates (0.22, 0.25, and 0.39 for 2017, 2018, and 2019, respectively) did not attain the standard of one per 100,000 population less than 15 years of age during the surveillance period [5,27]. Continuation of the every-four-week ES sampling from multiple watersheds in four of Haïti’s large population centers, with ongoing site evaluation, is necessary for the foreseeable future, at least for several years after the cessation of OPV use [3].

## Figures and Tables

**Table 1:** Haïti poliovirus environmental surveillance sites evaluated during March 2017–December 2019; site details, including estimated watershed populations. Port au Prince: BNF = Bois de Neuf, BDC = Bois de Chêne, MAC = Morne à Cabri, RRD = Route Rails Diquini, CAC = Cite au Cayes, CAR = Carrefour; Gonaïves: KHF = Key Soleil Health Facility, KSB = Key Soleil Bridge, ATP = Autorité Portuaire, ALL = Avenue Leon Legros, BRA = Boulevard de l’Avenir, KHS = Key Soleil School; Saint Marc: AMA = Avenue Maurepas, HUC = Impasse Hucar, and PET = Rue Petion; Cap Haïtien: CRC = Ruelle Caporis, RPA = Ruelle Patience, GRC = Grand Rue Champin, IMP = Impasse Petion, RNB = Route National Bridge, and CMB = Rivière Commerce Bridge.

City	Site Code	Total Sampling Events	Geographic Coordinates	Estimated Watershed Population	Additional Site Details
Port au Prince	BNF	34	18.5815, −72.3291	347,237 *	Downstream from CAC [17]
	BDC	34	18.5383, −72.3539	339,624 *	[17]
	MAC	1	18.6611, −72.1870	Unknown †	[17]
	RRD	33	18.5345, −72.3843	67,320	Open canal near intersection Rue du Dr Dehoux and Harry Truman Blvd
	CAC	4	18.5658, −72.3105	139,987	Upstream from BNF, open canal Saint George
	CAR	4	18.5500, −72.4166	9361	Open canal Rivière Froide on Route de Rails
Gonaïves	KHF	16	19.4534, −72.6900	20,749 *	[17]
	KSB	5	19.4575, −72.6960	82,123 *	[17]
	ATP	1	19.4483, −72.6963	Unknown †	[17]
	ALL	4	19.4422, −72.6845	10,034	Upstream from BRA, open canal near intersection of Ave Leon Legros and Ruelle Patience
	BRA	31	19.4383, −72.6896	20,241	Downstream from ALL, open canal near intersection Blvd de l’Avenir and Bas Mavignole
	KHS	4	19.4532, −72.6849	17,878	Upstream from KHF, open canal near intersection Avenue Roland 1 and Rue Polkos
Saint Marc	AMA	19	19.1059, −72.7022	50,744	Open canal at intersection Rivière Saint Marc and Avenue Maurepas ‡
	HUC	16	19.1224, −72.6978	5655	Open canal near intersection Impasse Hucar and Rue les Boutin
	PET	6	19.1102, −72.6984	49,372	Open canal at intersection Rivière Saint Marc and Rue Petion ‡
Cap Haïtien	CRC	21	19.7336, −72.2178	8810	Open canal along Ruelle Caporis, drains into Rivière du Haut du Cap
	RPA	21	19.7383, −72.1844	22,468	Open canal along Ruelle Patience
	GRC	3	19.7367, −72.2155	5455	Open canal along Grand Rue (Ruelle) Champin, drains into Rivière du Haut du Cap
	IMP	5	19.7405, −72.2088	9626	Open canal along footpath south of Impasse Petion, drains into Rivière du Haut du Cap
	RNB	5	19.7504, −72.2046	225,762	Site is Rivière du Haut du Cap under the Route National 3 bridge
	CMB	5	19.7336, −72.1772	21,580	Open canal adjacent to the Voix Evangélique d’Haïti (4VEH) radio transmission station

* Updated from previously published figures [17]; † unknown [17]; ‡ based on satellite imagery, there is no overlap between these two tributaries of the Rivière Saint Marc that flow through the city of Saint Marc [24].

**Table 2:** Summary of enterovirus isolation for samples collected through Haïti poliovirus environmental surveillance during March 2016–December 2019 and considerations for retaining or terminating sampling sites. Port au Prince: BNF = Bois de Neuf, BDC = Bois de Chêne, MAC = Morne à Cabri, RRD = Route Rails Diquini, CAC = Cite au Cayes, CAR = Carrefour. Gonaïves: KHF = Key Soleil Health Facility, KSB = Key Soleil Bridge, ATP = Autorité Portuaire, ALL = Avenue Leon Legros, BRA = Boulevard de l’Avenir, KHS = Key Soleil School. Saint Marc: AMA = Avenue Maurepas, HUC = Impasse Hucar, and PET = Rue Petion. Cap Haïtien: CRC = Ruelle Caporis, RPA = Ruelle Patience, GRC = Grand Rue Champin, IMP = Impasse Petion, RNB = Route National Bridge, and CMB = Rivière Commerce Bridge. SL = Sabin-like poliovirus.

City	Site Code	Total Sampling Events (% enterovirus Isolation) Mar 2017–Dec 2019	Total Sampling Events (% enterovirus Isolation) Mar 2016– Feb 2017 [17]	Estimated Watershed Population	Additional Considerations	Status
Port au Prince	BNF	34 (94)	12 (100)	347,237 †	Periodic SL poliovirus isolation; represents a watershed of northern Port au Prince	Retained
	BDC	34 (94)	12 (92)	339,624 †	Periodic SL poliovirus isolation; represents a watershed between BNF and RRD	Retained
	MAC	1 (100)	12 (75)	Unknown ‡	High levels of particulate matter in samples; population represented was unknown	Terminated
	RRD	33 (91)	N/A	67,321	Periodic SL poliovirus isolation; represents a watershed of southern Port au Prince	Retained
	CAC	4 (100)	N/A	139,987	Upstream from BNF; smaller watershed population than BNF	Terminated
	CAR	4 (50)	N/A	9361	Very small estimated watershed population	Terminated
Gonaïves	KHF	16 (50)	12 (58)	20,749 †	§	Terminated
	KSB	5 (0)	12 (25)	82,123 †	§	Terminated
	ATP	1 (0)	12 (0)	Unknown ‡	§	Terminated
	ALL	4 (50)	N/A	10,034	Upstream from BRA; smaller watershed population than BRA	Terminated
	BRA	31 (84)	N/A	20,241	Periodic SL poliovirus isolation; downstream from ALL; larger watershed population than ALL	Retained
	KHS	4 (0)	N/A	17,878	§	Terminated
Saint Marc	AMA	19 (90)	N/A	50,744	Periodic SL poliovirus isolation; represents a watershed of southern Saint Marc	Retained
	HUC	16 (50)	N/A	5655	Canal was periodically dry	Terminated
	PET	6 (67)	N/A	49,372	Wide canal with large volume of water; represents a watershed between AMA and HUC	Retained
Cap Haïtien	CRC	21 (81)	N/A	8810	Periodic SL poliovirus isolation, represents a watershed of western Cap Haïtien	Retained
	RPA	21 (67)	N/A	22,468	Periodic SL poliovirus isolation, represents a watershed of eastern Cap Haïtien	Retained
	GRC	3 (100)	N/A	5455	Very small estimated watershed population	Terminated
	IMP	5 (100)	N/A	9626	Represents a watershed of western Cap Haïtien	Retained
	RNB	5 (20)	N/A	225,762	Possible influence of high salinity due to proximity to sea	Terminated
	CMB	5 (80)	N/A	21,580	Represents a watershed of eastern Cap Haïtien	Retained

N/A = not applicable, site did not exist during surveillance period; † updated from previously published figures [17]; ‡ unknown [17]; § no additional considerations beyond enterovirus isolation.

## Data Availability

Not applicable.

## Supplementary Materials

The following are available online at https://www.mdpi.com/1999-4915/13/3/505/s1, Table S1: Selected water quality measurements, time of day of collection, and general weather conditions at sites for samples collected through Haïti poliovirus environmental surveillance during March 2017–December 2019. Figure S1: Enteroviruses isolated through Haïti poliovirus environmental surveillance during March 2017–December 2019 by year, epidemiological week, sampling site, and processing method.
