# Supplementary material for: Environmental Surveillance for Polioviruses in Haïti (2017–2019): The Dynamic Process for the Establishment and Monitoring of Sampling Sites
Source: Viruses. 2021 Mar 18;13(3):505. doi: 10.3390/v13030505 (PMC8003210; doi:10.3390/v13030505)
Supplement: Supplementary file 1 [file viruses-13-00505-s001.pdf]

Table S1. Selected water quality measurements, time of day of collection, and general weather conditions at sites for samples collected through Haïti poliovirus environmental surveillance during March 2017 – December 2019. **Port au Prince:** BNF = Bois de Neuf, BDC = Bois de Chêne, MAC = Morne à Cabri, RRD = Route Rails Diquini, CAC = Cite au Cayes, CAR = Carrefour; **Gonaïves:** KHF = Key Soleil Health Facility, KSB = Key Soleil Bridge, ATP = Autorité Portuaire, ALL = Avenue Leon Legros, BRA = Boulevard de l'Avenir, KHS = Key Soleil School; **Saint Marc**: AMA = Avenue Maurepas, HUC = Impasse Hucar, and PET = Rue Petion; **Cap Haïtien:** CRC = Ruelle Caporis, RPA = Ruelle Patience, GRC = Grand Rue Champin, IMP = Impasse Petion, RNB = Route National Bridge, and CMB = Rivière Commerce Bridge.

City	Site Code	Total Sampling Events	Sample Temperature (ºC)		Laboratory-measured pH		Sample Collection Time (hour:minute, AM or PM)		Percent of Sampling Events with Rain on Day Prior to Collection		Percent of Sampling Events with Rain on Day of Collection	
			No. samples with data	Median	No. samples with data	Median	No. samples with data	Median	No. samples with data	No. (%) with rain	No. samples with data	No. (%) with rain
Port au Prince	BNF	34	24	28.2	32	7.0	31	8:18 AM	30	8 (27)	29	1 (3)
	BDC	34	27	29.4	32	7.0	31	8:56 AM	29	6 (21)	29	1 (3)
	MAC	1	1	32.2	1	7.2	1	12:12 PM	1	0 (0)	1	0 (0)
	RRD	33	25	27.6	28	6.8	31	9:28 AM	26	8 (31)	26	2 (8)
	CAC	4	3	29.4	4	7.0	3	10:26 AM	3	1 (33)	3	0 (0)
	CAR	4	4	28.6	4	6.6	4	12:13 PM	4	2 (50)	4	0 (0)
Gonaïves	KHF	16	14	27.2	13	7.1	15	8:28 AM	15	2 (13)	15	0 (0)
	KSB	5	5	32.5	3	7.0	5	1:21PM	5	1 (20)	5	0 (0)
	ATP	1	1	28.2	0	N/A	1	2:08 PM	1	0 (0)	1	0 (0)
	ALL	4	4	30.6	3	7.0	4	11:37	4	1 (25)	4	0 (0)
	BRA	31	23	26.4	27	7.2	28	7:20 AM	27	2 (7)	27	0 (0)
	KHS	4	4	32.8	2	7.0	3	1:05 PM	4	1 (25)	4	0 (0)
Saint Marc	AMA	19	14	28.5	18	7.0	18	8:50 AM	16	2 (13)	16	0 (0)
	HUC	16	13	27.6	16	7.0	16	8:30 AM	12	2 (17)	12	0 (0)
	PET	6	4	28.6	7	7.0	5	8:50 AM	5	1 (20)	5	0 (0)
Cap Haïtien	CRC	21	15	28.3	21	7.0	20	8:22 AM	18	1 (6)	19	1 (5)
	RPA	21	15	27.3	21	7.0	20	7:24 AM	18	1 (6)	17	1 (6)
	GRC	3	3	28.0	3	7.0	3	8:48 AM	3	0 (0)	3	0 (0)
	IMP	5	2	31.5	5	7.4	4	9:42 AM	4	0 (0)	4	0 (0)
	RNB	5	1	29.9	5	7.0	4	8:34 AM	3	0 (0)	4	0 (0)
	CMB	5	2	30.8	5	7.0	4	9:01 AM	3	0 (0)	3	0 (0)

N/A: Data not available

Figure S1. Enteroviruses isolated through Haïti poliovirus environmental surveillance during March 2017 – December 2019 by year, epidemiological week, sampling site, and processing method. Orange-shading for months in 2018 and May 2019 indicates periods of tetanus-diphtheria outbreak response campaigns in certain regions that provided an opportunity for intensified all-antigen vaccination and for July – September 2019 indicates the approximate time period of a nation-wide supplementary immunization activity targeting children 2-59 months of age with bivalent oral polio vaccine (bOPV, Sabin strain serotypes 1 and 3 containing). NPEV = non-polio enterovirus; SL1 = Sabin-like poliovirus of serotype 1; SL3 = Sabin-like poliovirus of serotype 3; TP = Two phase processing method; CA = CaFÉ processing method.

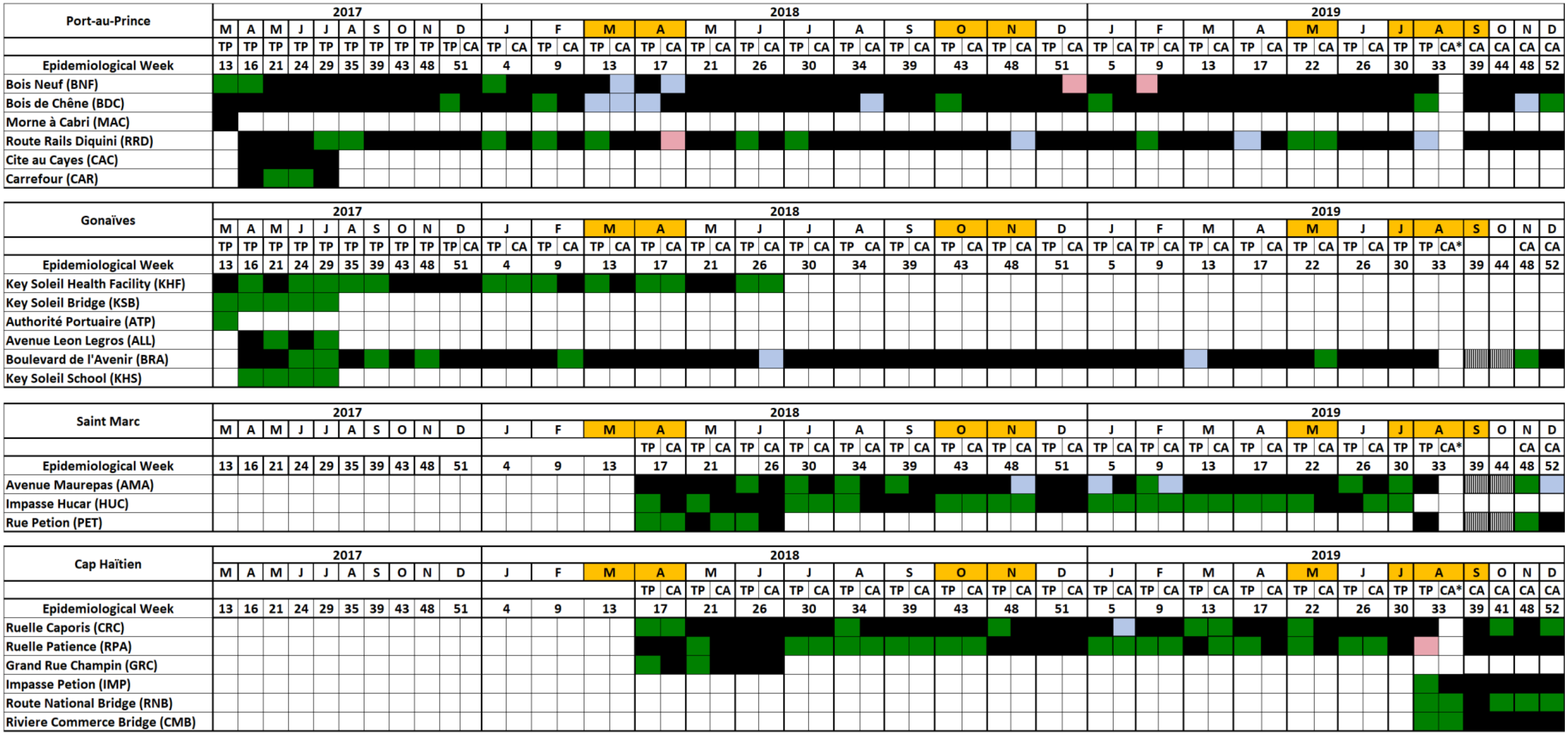

TP = Two-phase separation method; CA = Concentration and Filter Elution filtration method (CaFÉ); CA* = only selected samples processed using CA method this month.

Collection not scheduled or prcessing method not used

Negative

NPEV

SL1 +/- NPEV

SL3 +/- NPEV

Collection not conducted due to civil insecurity
